# Factors associated with intubation and heated high-flow nasal cannula use in hospitalized respiratory syncytial virus infected children: A single-center retrospective cohort study

**DOI:** 10.1371/journal.pone.0327541

**Published:** 2025-08-07

**Authors:** Nichaphat Keelapang, Kanokkarn Sunkonkit

**Affiliations:** 1 Department of Pediatrics, Faculty of Medicine, Chiang Mai University, Chiang Mai, Thailand; 2 Department of Pediatrics, Division of Pulmonary and Sleep Medicine, Faculty of Medicine, Chiang Mai University, Chiang Mai, Thailand; Universitair Kinderziekenhuis Koningin Fabiola: Hopital Universitaire des Enfants Reine Fabiola, BELGIUM

## Abstract

**Background:**

Respiratory syncytial virus (RSV) is a leading cause of severe lower respiratory tract illness (LRTI) in children, often requiring hospitalization and respiratory support. This study, therefore, aims to identify factors associated with intubation and heated high-flow nasal cannula (HHFNC) use in children hospitalized with RSV infection.

**Methods:**

This retrospective study reviewed medical records of children aged 0 month to 15 years hospitalized with RSV infection at Chiang Mai University Hospital between January 2018 and December 2022. Baseline characteristics, clinical features, and laboratory findings were analyzed.

Factors associated with intubation or HHFNC use were analyzed using univariable and multivariable logistic regression with significance set at p < 0.05.

**Result:**

Among 260 children (53.8% male; median age 28 months, IQR 12–44), 76.5% required low-flow oxygen therapy, 11.5% required HHFNC, and 11.9% required intubation, respectively.

Prematurity (22.7%) and respiratory comorbidities (17.6%) were common. HHFNC use was significantly associated with prematurity (adjusted odds ratio [aOR] 3.11, p = 0.016), chest retractions (aOR 5.42, *p = 0.017*), and multi-lobar infiltrates on chest X-ray (aOR 7.52, *p < 0.001*). Factors associated with intubation included age ≤ 2 years (aOR 3.70, *p = 0.008*), prematurity (aOR 5.68, *p < 0.001*), chest retractions (aOR 4.39, *p = 0.033*), and multi-lobar infiltrates (aOR 8.83, *p < 0.001*).

**Conclusions:**

Prematurity, younger age, chest retractions, and multi-lobar infiltrates on chest X-ray were key predictors for HHFNC and intubation in RSV-infected children. These findings may inform risk stratification and management strategies for severe RSV-related illness in pediatric patients.

## Introduction

Respiratory Syncytial Virus (RSV) is a leading cause of acute lower respiratory tract infections (ALRTIs) in young children, with significant morbidity, mortality, and high healthcare resource demands [1–3]. Infants—especially those born prematurely or with underlying heart or lung conditions—are at heightened risk for severe RSV, leading to frequent hospitalizations and intensive care needs, especially during colder seasons. Globally, RSV accounts for about 3 million hospital admissions and over 100,000 deaths annually in children under five, imposing considerable costs and straining healthcare systems, particularly in low-resource settings [3]. Additionally, severe RSV in infancy is linked to a higher risk of recurrent wheezing and childhood asthma [4].

In Thailand, RSV is a leading cause of respiratory infections in children under five, with infants most at risk [5–7]. The RSV season coincides with the rainy months (August to November) [5,6], differing from temperate regions. In 2020, the incidence of RSV-associated lower respiratory tract infections (LRTI) reached 3.96 per 100,000 children, with pneumonia as the primary cause of mortality [5]. Similar to global trends, RSV drives significant pediatric hospitalizations in Thailand, especially among children under two and those with comorbidities such as prematurity, chronic lung disease, congenital heart disease, or immunosuppression [8–10]. During peak season, the demand for respiratory support escalates, often requiring heated high-flow nasal cannula (HHFNC) or mechanical ventilation in severe cases [9]. These needs strain healthcare resources, particularly in settings with limited pediatric ICU capacity [5,9,11–13]. However, despite this substantial burden, data on predictors for HHFNC use and intubation in Thai children with RSV are limited. Most evidence comes from international studies, which may not fully reflect local clinical profiles and healthcare constraints.

Management of RSV-associated LRTI relies on supportive care, including hydration, nutritional support, and continuous monitoring of oxygenation and vital signs [14,15]. Respiratory support varies by severity: low-flow nasal cannula is commonly used in mild to moderate cases, while HHFNC is increasingly preferred in more severe cases to improve oxygenation and potentially prevent the need for invasive ventilation [14,16]. Intubation and mechanical ventilation are reserved for children with severe respiratory failure unresponsive to non-invasive support [16]. Early identification of children likely to require HHFNC or intubation is crucial to optimize care and resource allocation.

Risk factors for severe RSV-LRTI include prematurity [6,17,18], young age (<3 months) [8,13,18], bacterial co-infection [19–21], and underlying comorbidities such as congenital heart disease, neuromuscular disorders, and immunosuppression [5,9,17,18]. In Thailand, while all hospitalized children with RSV-LRTI require oxygen therapy, many progress to advanced respiratory support, including HHFNC or mechanical ventilation. However, the predictors of escalation to HHFNC or intubation remain unclear, and the relationship between these interventions is not well-defined in the Thai context. Existing studies are largely based on international data, which may not reflect local clinical practices or healthcare resource constraints. This study aims to fill this gap by examining the incidence and associated factors for HHFNC use and intubation among children hospitalized with RSV in a tertiary care center in Thailand, with the goal of informing early risk stratification and improving clinical management.

## Materials and methods

### Study design

We conducted a retrospective observational study at Chiang Mai University Hospital. The study population comprised children aged 0–15 years who were admitted to CMU Hospital from January 2018 to December 2022 with confirmed RSV infection via nasopharyngeal swab. This study received ethical approval from the Research Ethics Committee, Faculty of Medicine, Chiang Mai University (Study code: PED-2566–0244). Informed consent was waived due to minimal risk, and the data were analyzed anonymously. The data were accessed for research purpose during October 1, 2023-October 31,2024.

### Patient population

Inclusion criteria was pediatric patients aged 0 month to 15 years of age who were admitted to CMU Hospital between January 2018 and December 2022 with confirmed RSV infection, as identified through nasopharyngeal swab testing recorded in the hospital’s laboratory database. No exclusion criteria were applied.

### Data collection

All data for this study were retrospectively reviewed from hospital medical records. Collected demographic information included admission date, age, gender, ethnicity, gestational age (GA), use of postnatal oxygen or positive pressure ventilation, and any underlying conditions (such as bronchopulmonary dysplasia (BPD), asthma or allergic rhinitis, congenital heart disease, neurological, hematologic, gastrointestinal disorders, or other diseases). Additional information included bedridden status, breastfeeding history and duration, daycare attendance, exposure to second-hand smoke, and vaccination history. Initial clinical presentation details were recorded, including vital signs, oxygen saturation, and chest examination findings (e.g., retractions, accessory muscle use, air entry, and adventitious sounds) within the first 24 hours after admission. Initial laboratory results and chest X-ray findings obtained within the first 24 hours of admission were recorded. Respiratory support interventions, including low-flow oxygen—defined as supplemental oxygen delivered via nasal cannula or simple face mask at age-appropriate flow rates that do not generate positive airway pressure, HHFNC, and mechanical ventilation, were documented. For the purpose of analysis, patients were categorized according to the highest level of respiratory support received within the first 24 hours of hospitalization to reflect the peak severity of respiratory compromise during the early phase of illness. Data on adjunctive treatments such as nebulization, systemic corticosteroid administration, and empirical antibiotic use were also collected. Additionally, information on the duration of intubation, length of ICU stay, total hospital stay, and the ROX index—calculated as the ratio of oxygen saturation to the fraction of inspired oxygen and respiratory rate (SpO_2_/FiO_2_/RR) during HHFNC use—was included.

#### Definitions and clarifications.

**Gestational Age:** The gestational age reported in our dataset refers to the gestational age at birth. This information was abstracted from the birth history section of the medical records.

**Bedridden Status:** The term “bedridden” was used to describe patients with significant neurodevelopmental impairments or chronic neurological disorders who were non-ambulatory, dependent on caregivers for all activities of daily living, and required full-time support.

**Interpretation of Chest Radiographic Findings:** Chest X-ray findings were reviewed and interpreted by a board-certified pediatric pulmonologist (KS) as part of routine clinical care. The radiographic features assessed included the presence of hyperinflation, peribronchial thickening, focal or multilobar infiltrates, and atelectasis. Findings were recorded from the initial chest radiograph obtained upon admission.

**Low-Flow Oxygen:** Low-flow oxygen was defined as supplemental oxygen administered via nasal cannula or simple face mask at flow rates appropriate for age, which do not provide positive airway pressure.

**Criteria for Initiation of HHFNC and Invasive Ventilation** Decisions regarding escalation of respiratory support were made at the discretion of the attending pediatrician or pediatric intensivist. The initiation of HHFNC or invasive mechanical ventilation was typically based on clinical indicators of respiratory distress, such as tachypnea, subcostal or intercostal chest retractions, nasal flaring, hypoxemia despite low-flow oxygen therapy, and signs of respiratory fatigue or altered mental status. These criteria align with standard clinical practice in our institution, although specific institutional protocols or scoring systems were not formally applied during the study period. However, non-invasive ventilation modalities, including continuous positive airway pressure (CPAP) and bi-level positive airway pressure (BiPAP) were not routinely employed in our pediatric general ward or PICU during the study period due to equipment limitations and institutional practice preferences. As such, no patients in our cohort received NIV.

**Classification of Respiratory Support Groups** Patients were categorized according to the highest level of respiratory support received within the first 24 hours of hospitalization. This classification was used to capture the severity of respiratory illness during the early phase of admission. Specifically:

1) Children who received low-flow oxygen only were assigned to the “low-flow oxygen” group.
2) Those who required HHFNC but did not undergo intubation were categorized under the “HHFNC” group.
3) Patients who progressed to intubation, regardless of whether they initially received low-flow oxygen or HHFNC, were classified in the “intubation” group.
4) Patients who received both HHFNC and intubation were thus included in the “intubation” group to reflect the highest level of support required.

**HFNC failure**: HFNC failure was defined as the requirement for escalation to invasive mechanical ventilation following the initiation of high-flow nasal cannula therapy. This determination was made based on the clinical judgment of the treating physician. Common criteria for escalation included persistent or worsening signs of respiratory distress, hypoxemia refractory to HFNC, rising carbon dioxide levels (hypercapnia), and clinical indicators of fatigue or altered mental status.

### Statistical analysis

Data analysis was conducted using IBM SPSS version 29.0. Descriptive statistics were used to evaluate overall demographic data, initial clinical presentation, treatment information, and outcomes throughout the hospital stay. Comparisons were made between groups receiving low-flow nasal cannula, HHFNC, and intubation. For continuous variables, comparisons between groups were made using the Student’s t-test for normally distributed data and the Mann-Whitney U-test for skewed data. Categorical variables were assessed with the Chi-square test or Fisher’s exact test. Statistical significance was set at a p-value of less than 0.05. Factors associated with the use of HHFNC or intubation were analyzed through univariable and multivariable logistic regression models.

To enhance the reliability of data in this retrospective study, we included only patients with laboratory-confirmed RSV infection and complete hospitalization records. Variables with more than 50% missing data were excluded from the final analysis to mitigate the risk of bias due to incomplete information. Specifically, data points such as breastfeeding for at least 6 months, daycare attendance, and exposure to second hand smoke were excluded from multivariable analyses due to their high proportion of missing values.

Variables for inclusion in the multivariable logistic regression model were selected based on a combination of prior knowledge, clinical expertise, and findings from a comprehensive literature review. Specifically, we prioritized variables recognized as clinically relevant risk factors for severe RSV infection including age, prematurity, underlying chronic medical conditions, and clinical severity indicators at admission. This strategy was employed to ensure the inclusion of meaningful predictors while minimizing the risk of overfitting, given the limited number of events in some subgroups.

## Result

### Baseline characteristics

A total of 260 pediatric patients hospitalized with RSV infection at Chiang Mai University Hospital were included in this study. At the time of admission, 212 patients received low-flow oxygen therapy, 37 received HHFNC, and 11 were intubated. Within the first 24 hours of hospitalization, 13 of the 212 patients initially managed with low-flow oxygen required escalation to HHFNC. Among the 37 children who started with HHFNC, 20 subsequently required intubation due to clinical deterioration. Ultimately, 199 patients (76.5%) were managed with low-flow oxygen only, 30 (11.5%) received HHFNC (either initially or after escalation), and 31 (11.9%) underwent mechanical ventilation. The median age was 28 ± 16 months, and 53.8% were male. A history of preterm birth (GA < 37 weeks) was documented in 59 patients (22.7%), including 11 very preterm cases. Postnatal oxygen use was reported in 63 patients (24.2%), with 18.1% having required intubation or non-invasive ventilation during the neonatal period. Common comorbidities included BPD or chronic lung disease (8.5%), asthma or allergic rhinitis (8.9%), and congenital heart disease (15%), respectively. The predominant clinical presentations were cough (91%), rhinorrhea (70%), and dyspnea (55%). The most frequent chest X-ray findings included perihilar infiltrates and/or hyperinflation (49.6%). Empirical antibiotics were prescribed in 65% of cases, primarily third-generation cephalosporins (34%), azithromycin (21.5%), and levofloxacin (8%). Systemic corticosteroids were used in 39%. Antibiotic and corticosteroid use was higher in non-low-flow oxygen groups. The overall median hospital stay was 6.0 days (IQR 4.0–13.8), with longer stays in the intubation group (18 days) compared to HHFNC (9 days) and low-flow oxygen therapy groups (8 days). Patient characteristics are detailed in Table 1.

**Table 1:** Baseline characteristics of hospitalized children with RSV (n = 260).

Parameters		Low flow oxygen (n = 199)	HHFNC (n = 30)	Intubation (n = 31)	Total (n = 260)
**Age (months)**		30.0 (14.0, 46.0)	31.0 (8.5, 50.0)	15.0 (5.0, 29.0)	28.0 (12.0,44.0)
**Male, n (%)**		113 (56.8)	15 (50)	12 (38.7)	140 (53.8)
**Gestational age (week)**	**≥ 37 week, n (%)**	168 (84.4)	18 (60)	15 (48.4)	201 (77.3)
	**28-36 + 6 week, n (%)**	25 (12.6)	11 (36.7)	12 (38.7)	48 (18.5)
	**< 28 week, n (%)**	6 (3.0)	1 (3.3)	4 (12.9)	11 (4.2)
**Postnatal MV or NIV use, n (%)**		26 (13.1)	9 (30)	12 (38.7)	47 (18.1)
**Postnatal oxygen use, n (%)**		36 (18.1)	12 (40)	15 (48.4)	63 (24.2)
**Comorbidity**	**BPD/ chronic lung disease, n (%)**	12 (6.0)	4 (13.3)	6 (19.4)	22 (8.5)
	**Asthma, n (%)**	13 (6.6)	8 (26.7)	2 (6.5)	23 (8.9)
	**Congenital heart disease, n (%)**	27 (13.6)	5 (16.7)	7 (22.6)	39 (15)
	**Neurological disease, n (%)**	16 (8.0)	6 (20)	6 (19.4)	28 (10.8)
	**Hematological disease, n (%)**	36 (18.1)	3 (10)	3 (9.7)	42 (16.2)
	**Gastrointestinal disease, n (%)**	10 (5.0)	5 (16.7)	5 (16.1)	20 (7.7)
**Bedridden status, n (%)**		2 (1.0)	4 (13.3)	2 (6.5)	8 (3.1)
**Breastfeeding at least 6 months, n (%)**		42 (21.1)	7 (23.3)	9 (29.0)	58 (22.3)
**Daycare attendance, n (%)**		80 (40.2)	8 (26.7)	7 (22.6)	95 (36.5)
**Expose to second hand smoke, n (%)**		29 (14.6)	7 (23.3)	7 (22.6)	43 (16.5)
**Clinical presentation**	**Cough, n (%)**	181 (91)	29 (96.7)	27 (87.1)	237 (91.2)
	**Rhinorrhea, n (%)**	151 (75.9)	16 (53.3)	15 (48.4)	182 (70)
	**Wheezing, n (%)**	5 (2.5)	0 (0)	1 (3.2)	6 (2.3)
	**Dyspnea, n (%)**	93 (46.7)	25 (83.3)	24 (77.4)	142 (54.6)
	**Decreased appetite, n (%)**	74 (37.2)	14 (46.7)	7 (23.3)	95 (36.7)
**Systemic corticosteroids, n (%)**		62 (32.5)	21 (70)	15 (50)	98 (39)
**Empirical antibiotics**	**Third-generation cephalosporin, n (%)**	46 (23.1)	23 (76.7)	19 (61.3)	88 (33.8)
	**Azithromycin, n (%)**	42 (21.1)	7 (23.3)	7 (22.6)	56 (21.5)
	**Levofloxacin, n (%)**	13 (6.5)	6 (20.0)	2 (6.5)	21 (8.1)
**Chest X-ray**	**Atelectasis, n (%)**	36 (18.1)	6 (20.0)	12 (38.7)	54 (20.8)
	**Patchy infiltration, n (%)**	28 (14.1)	7 (23.3)	7 (22.6)	42 (16.2)
	**Perihilar/hyperinflation, n (%)**	120 (60.3)	8 (26.7)	1 (3.2)	129 (49.6)
	**Multi-lobar infiltration, n (%)**	15 (7.5)	9 (30.0)	11 (35.5)	35 (13.5)
**Length of hospital stay (days)**		5.0 (3.0,9.0)	9.0 (6.8, 15.0)	18.0 (11.0, 32.0)	6.0 (4.0,13.8)

*Low-flow oxygen—defined as supplemental oxygen delivered via nasal cannula or simple face mask at age-appropriate flow rates that do not generate positive airway pressure.*

### Logistic regression analysis for HHFNC use

The univariable and multivariable logistic regression analyses identified three significant risk factors for HHFNC use compared to low-flow oxygen therapy in RSV-infected children: a history of prematurity [aOR 3.36, 95% CI 1.12–10.03, *p = 0.030*], the presence of chest retractions [aOR 5.39, 95% CI 1.36–21.28, *p = 0.016*], and multilobar infiltrates on chest X-ray [aOR 7.69, 95% CI 2.42–24.48, *p < 0.001*] (Table 2).

**Table 2:** Factors associated with HHFNC Use in RSV infected children (n = 229).

Parameters	Low flow oxygen (n = 199)	HHFNC (n = 30)	Univariable analysis		Multivariable analysis	
			OR	p-value	aOR	p-value
**Age ≤ 2 years**	75 (37.7)	12 (40.0)	1.10 (0.50-2.42)	0.80	1.82 (0.71-4.68)	0.21
**Male, n (%)**	113 (56.8)	15 (50.0)	0.76 (0.35-1.64)	0.49	0.79 (0.33-1.87)	0.59
**Preterm GA < 37 weeks, n (%)**	31 (15.6)	12 (40.0)	3.61 (1.58-8.24)	*0.002* ^*^	3.36 (1.12-10.03)	*0.030* ^*^
**BPD/ chronic lung disease, n (%)**	12 (6.0)	4 (13.3)	2.39 (0.72-7.98)	0.15	0.81 (0.17-3.81)	0.79
**Congenital heart disease, n (%)**	27 (13.6)	5 (16.7)	1.27 (0.45-3.61)	0.65	1.68 (0.47-6.03)	0.42
**Retraction, n (%)**	116 (58.3)	27 (90.0)	6.44 (1.89-21.93)	*0.003* ^*^	5.39 (1.36-21.28)	*0.016* ^*^
**Fair/poor air entry, n (%)**	39 (19.6)	14 (46.7)	3.59 (1.61-7.97)	*0.002*	2.48 (0.95-6.44)	0.06
**Chest X-ray multi-lobar infiltration, n (%)**	15 (7.5)	9 (30.0)	5.26 (2.05-13.48)	*<0.001* ^*^	7.69 (2.42-24.48)	*<0.001* ^*^

*Statistical significance p-value < 0.05; low-flow oxygen—defined as supplemental oxygen delivered via nasal cannula or simple face mask at age-appropriate flow rates that do not generate positive airway pressure.

### Logistic regression analysis for intubation

Additionally, younger age (<2 years) [aOR 3.79, 95% CI 1.46–9.86, *p = 0.006*], a history of prematurity [aOR 5.64, 95% CI 1.85–17.22, *p = 0.002*], the presence of chest retractions [aOR 4.53, 95% CI 1.20–17.11, *p = 0.026*], and multilobar infiltrates on chest X-ray [aOR 9.26, 95% CI 2.84–30.23, *p < 0.001*] were identified as significant risk factors for intubation compared to low-flow oxygen therapy. Further details are presented in Table 3.

**Table 3:** Factors associated with intubation in RSV infected children (n = 230).

Parameters	Low flow oxygen (n = 199)	Intubation (n = 31)	Univariable analysis		Multivariable analysis	
			OR	p-value	aOR	p-value
**Age ≤ 2 years**	75 (37.7)	22 (71.0)	4.04 (1.77-9.24)	*<0.001* ^*^	3.79 (1.46-9.86)	*0.006* ^*^
**Male, n (%)**	113 (56.8)	12 (38.7)	0.64 (0.22-1.04)	0.06	0.45 (0.18-1.15)	0.09
**Preterm GA < 37 weeks, n (%)**	31 (15.6)	16 (51.6)	5.78 (2.59-12.89)	*<0.001* ^*^	5.64 (1.85-17.22)	0.002*
**BPD/ chronic lung disease, n (%)**	12 (6.0)	6 (19.4)	3.74 (1.29-10.85)	*0.015* ^*^	0.91 (0.21-3.92)	0.90
**Congenital heart disease, n (%)**	27 (13.6)	7 (22.6)	1.86 (0.73-4.73)	0.19	2.22 (0.65-7.52)	0.19
**Retraction, n (%)**	116 (58.3)	27 (87.1)	4.83 (1.63-14.32)	*0.005* ^*^	4.53 (1.20-17.11)	*0.026* ^*^
**Fair/poor air entry, n (%)**	39 (19.6)	11 (35.5)	2.25 (0.99-5.09)	0.50	1.95 (0.69-5.49)	0.21
**Chest X-ray multi-lobar infiltration, n (%)**	15 (7.5)	11 (35.5)	6.74 (2.73-16.67)	*<0.001* ^*^	9.26 (2.84-30.23)	*<0.001* ^*^

*Statistical significance p-value < 0.05; low-flow oxygen—defined as supplemental oxygen delivered via nasal cannula or simple face mask at age-appropriate flow rates that do not generate positive airway pressure.

### Logistic regression analysis for factors associated with HHFNC failure

Our study further explored factors associated with HHFNC failure in RSV patients who received HHFNC therapy. Alterations in vital signs following HHFNC initiation, including respiratory rate, heart rate, and the ROX index, may have significant associations with HHFNC outcomes. A comprehensive summary of factors associated with HFNC failure in children with RSV infection is presented in S1 Table.

## Discussion

Our study highlights the substantial burden of RSV as a leading cause of severe LRTIs in children, often necessitating advanced respiratory support. We present a comprehensive characterization of pediatric RSV cases, including clinical features, laboratory and radiologic findings at presentation, treatment approaches—such as oxygen therapy—and length of hospitalization.

Our study identified younger age (<2 years), a history of prematurity, the presence of chest retractions, and multilobar infiltrates on chest X-ray as factors significantly associated with the use of HHFNC or mechanical ventilation in children with RSV infection. These findings align with previous studies that have reported similar risk factors for severe RSV infection [9,13,17,18]. The association between prematurity and severe RSV infection is well-documented, with premature infants often exhibiting impaired lung development and weaker immune responses, increasing their susceptibility to severe disease [22–24]. This finding also aligns with prior studies showing an elevated risk of hospitalization and respiratory support in this population [25–28].

Similarly, chest retractions, a clinical marker of respiratory distress, and multi-lobar infiltrates, indicative of extensive pulmonary involvement [25], were significant predictors of both HHFNC use and intubation. These results corroborate earlier reports that severe pulmonary involvement often necessitates more aggressive interventions. Several studies have identified underlying conditions, such as BPD and cardiovascular disease, as risk factors for severe RSV infection. However, in our study, these comorbidities were not significantly associated with the use of HHFNC or the need for intubation in RSV-infected children [17,18]. Although chest retractions are often a determinant in the clinical decision to initiate respiratory support, their association with higher levels of support remains clinically relevant, as they may help identify patients who require closer monitoring and timely intervention, particularly in settings where objective severity scoring systems are not routinely implemented.

Additionally, our finding that younger age is independently associated with intubation risk is consistent with previous studies emphasizing the vulnerability of infants and toddlers to severe RSV-related complications [25,29]. Younger children face a heightened risk of intubation during respiratory infections due to their anatomical and physiological characteristics [30–32]. Their narrower airways are particularly susceptible to obstruction, as even minor inflammation or mucus accumulation can disproportionately increase airflow resistance and lead to respiratory distress [32]. Additionally, infants have a higher chest wall compliance and reduced functional residual capacity, which can contribute to rapid desaturation during respiratory compromise [30,32,33]. In addition, the immunological immaturity of neonates—characterized by attenuated type I interferon responses and a Th2-skewed adaptive profile—limits their capacity to mount effective defenses against respiratory pathogens [34]. This vulnerability contributes to the increased severity of infection observed in younger children, who are more likely to require PICU admission, prolonged hospitalization, and advanced respiratory support [35], including intubation [36].

Interestingly, approximately two-thirds of patients in our study were prescribed antibiotics, a proportion notably higher than the 37.9% intravenous antibiotic use reported by Aikphaibul et al. in their study of 427 hospitalized children with RSV-associated lower respiratory tract infections at a tertiary care center in Bangkok, Thailand [9]. Samson et al. reported that among RSV-infected patients receiving antibiotics, 32% had positive blood or urine cultures. Similarly, Thorburn et al. found that 42.4% of RSV bronchiolitis patients admitted to the PICU had positive bacterial cultures; however, half of these cases (20.4%) involved low bacterial growth, while the remaining 21.8% were confirmed bacterial co-infections (≥10^5^ CFU/mL). Numerous studies have highlighted the association between severe RSV infection and bacterial co-infections [20,21]. In our study, the decision to initiate antibiotic therapy was largely based on the clinical judgment of the treating physicians, who assessed the severity of the patient’s condition and the likelihood of bacterial co-infection. This underscores the importance of cautious and evidence-based antibiotic use in managing severe RSV infections.

BPD and congenital heart disease are well-established risk factors for severe RSV infection, as consistently demonstrated in the literature and incorporated into current immunoprophylaxis guidelines. In our study, multivariable analysis did not show statistically significant associations between BPD or congenital heart disease and the need for HHFNC or intubation; however, these findings should be interpreted with caution. Notably, the proportion of patients with BPD (19%) and congenital heart disease (22%) was higher among those who were intubated compared to the overall cohort (8% and 15%, respectively), suggesting a potential concentration of these comorbidities among patients with more severe disease.

The absence of statistical significance likely reflects limited power due to small subgroup sizes and event counts, as well as potential multicollinearity between prematurity and BPD—where adjustment for prematurity may have attenuated the independent association of BPD with severe outcomes. These findings should not be interpreted as contradicting the established role of BPD and congenital heart disease in RSV severity. Rather, they emphasize the need for cautious interpretation and support the necessity of larger, adequately powered studies to validate these associations. In Thailand, access to palivizumab prophylaxis only became available in 2024 and remains largely limited to self-paying families, as it is not yet integrated into national health policy. This restricted availability may have influenced the observed disease severity in our cohort and highlights the broader challenges of implementing RSV prevention strategies in resource-limited settings. Further prospective studies are warranted to clarify these associations and guide preventive policy in similar healthcare environments.

Although limited by a small sample size, our analysis of factors associated with HFNC failure in RSV-infected children provides clinically valuable insights. Early identification of patients at risk for HFNC failure is essential for guiding close monitoring and timely intervention. Prior studies have identified several predictors of HFNC failure in pediatric populations, including younger age, congenital heart disease, prior intubation, elevated clinical severity scores, increased oxygen requirements, lobar infiltrates, and a lack of early improvement in vital signs [37–39]. In contrast, our findings suggest that early reductions in heart rate and respiratory rate, along with an increase in the ROX index within the first two hours of HFNC initiation, are associated with treatment success. These observations are consistent with previous research [39–41] and emphasize the utility of early physiological changes as indicators of HFNC response. Continuous monitoring of these parameters may aid clinicians in distinguishing between responders and non-responders, thereby optimizing clinical decision-making in the management of RSV-infected children.

This study offers valuable insights into the predictors of respiratory support in RSV-infected children, leveraging a robust sample size from a tertiary care hospital in Thailand. However, several limitations warrant consideration. First, the retrospective design inherently introduces selection bias and limits the generalizability of our findings beyond similar tertiary care settings. The absence of specific exclusion criteria may have contributed to population heterogeneity; however, this approach was deliberate to reflect real-world clinical practice and to preserve external validity. By including all hospitalized children with confirmed RSV infection, we aimed to capture the full clinical spectrum and avoid selection bias. We acknowledge, however, that this inclusiveness may have introduced potential confounders, such as underlying comorbidities and variations in clinical management. To address this, we employed multivariable regression analyses adjusting for relevant baseline characteristics, including age, prematurity, chronic illnesses, and severity indicators at admission, to reduce residual confounding. Additionally, the relatively small number of cases in certain subgroups—particularly those requiring HHFNC or intubation—may have limited the statistical power to detect associations with less frequent predictors. Given the retrospective nature and the fixed sample size based on existing hospital records, a formal power calculation was not feasible. As such, these limitations should be considered when interpreting subgroup analyses and the generalizability of the findings. Second, data on key confounding factors, such as RSV subtypes and co-infections, were not included in the analysis. Third, treatment decisions were based on individual physician judgment, which could introduce variability. Fourth, the COVID-19 pandemic likely impacted RSV incidence during the study period, potentially influencing the results. Fifth, the single-center design may not capture regional or global differences in RSV epidemiology and management practices. Lastly, despite our efforts to include only complete hospitalization records, certain variables—such as breastfeeding duration, daycare attendance, and secondhand smoke exposure—had a substantial proportion of missing data and were therefore excluded from the final analysis. This exclusion may limit our ability to fully assess the influence of these factors on respiratory outcomes. Moreover, as a retrospective study, we were constrained by the accuracy and completeness of the existing medical records, which may have introduced information bias. We acknowledge these limitations and recommend that future prospective studies include comprehensive data collection on these potentially important exposures.

Despite these limitations, our findings offer meaningful clinical implications. The identification of specific risk factors—such as prematurity, pronounced respiratory distress, and radiologic evidence of multilobar involvement—provides actionable insights for early clinical decision-making. These predictors may support timely escalation of care, prioritization of monitoring, and optimal allocation of limited healthcare resources. Notably, the strong association between chest X-ray findings and the need for advanced respiratory support underscores the utility of imaging as a practical and accessible tool for risk stratification in RSV-infected children, particularly in resource-constrained settings. By emphasizing readily available clinical and radiologic indicators, our results contribute to improved triage strategies and care planning in settings where advanced diagnostics or intensive care capacity may be limited. These findings also underscore the importance of developing context-specific clinical protocols to guide respiratory support decisions. Future prospective studies are warranted to validate these associations in broader populations and to explore additional predictive factors—such as viral genotypes, co-infections, and inflammatory biomarkers. Moreover, evaluating the long-term outcomes of children who require HHFNC or intubation for RSV infection will be crucial for informing clinical guidelines and shaping targeted preventive measures, including the use of RSV immunoprophylaxis in high-risk pediatric populations.

The identification of key predictors—namely prematurity, chest retractions, and multilobar infiltrates—supports early risk stratification and informed planning for respiratory support in children hospitalized with RSV infection. Although these associations have been previously reported, our study provides further validation within the context of a tertiary care center in a middle-income Southeast Asian country. This region-specific evidence may be particularly relevant for healthcare systems facing similar epidemiological patterns and resource limitations. By confirming these predictors in our cohort, we underscore their applicability to clinical decision-making across diverse settings and support their integration into protocols for care prioritization and resource allocation in pediatric and intensive care units. Additionally, the high rate of antibiotic use observed—despite the viral nature of RSV—likely reflects clinical uncertainty or precautionary practices to prevent bacterial co-infection. This highlights the need for strengthened antimicrobial stewardship and further investigation into the prevalence and impact of bacterial co-infection in RSV-related hospitalizations.

## Conclusions

Our study identified younger age, prematurity, chest retractions, and multilobar infiltrates on chest radiography as significant predictors for the use of HHFNC or intubation among children hospitalized with RSV infection. These findings emphasize the importance of early risk recognition and proactive clinical management in vulnerable patients. Future prospective, multicenter studies are warranted to confirm these associations and enhance the generalizability of our results. Additionally, the integration of inflammatory biomarkers, immune response indicators, or RSV viral subtyping may further improve risk stratification and support the development of more personalized treatment strategies.

## Supporting information

S1 Table Factors associated with HFNC failure in RSV infected children. (PDF)

S2 File RSV_data_PlosOne_submission. (XLSX)
