# Supplementary material for: Factors associated with intubation and heated high-flow nasal cannula use in hospitalized respiratory syncytial virus infected children: A single-center retrospective cohort study
Source: PLoS One. 2025 Aug 7;20(8):e0327541. doi: 10.1371/journal.pone.0327541 (PMC12331089; doi:10.1371/journal.pone.0327541)
Supplement: S1 Table — (PDF) [file pone.0327541.s001.pdf]

Supplementary table 1 Factors associated with HFNC failure in RSV Infected Children (n =50)

Parameters	HFNC success (n=30)	HFNC failure (n=20)	Univariable analysis	
			OR	p-value
**Age ≤ 2 years**	12 (40.0)	11 (55.0)	1.83 (0.58-5.76)	0.29
**Male, n (%)**	15 (50.0)	8 (40.0)	0.67 (0.21-2.09)	0.49
**Preterm GA < 37 weeks, n (%)**	12 (40.0)	10 (50.0)	1.50 (0.48-4.69)	0.48
**BPD/ chronic lung disease, n (%)**	4 (13.3)	4 (20.0)	1.63 (0.36-7.42)	0.53
**Congenital heart disease, n (%)**	5 (16.7)	3 (15.0)	0.88 (0.19-4.19)	0.88
**Retraction, n (%)**	27 (90.0)	18 (90.0)	1.00 (0.15-6.59)	1.00
**Fair/poor air entry, n (%)**	14 (46.7)	8 (40.0)	0.76 (0.24-2.39)	0.64
**Chest X-ray multi-lobar infiltration, n (%)**	9 (30.0)	6 (30.0)	1.00 (0.29-3.43)	1.00
**Respiratory rate at 0 hour (/min)$**	48 (40, 57)	50 (41, 60)	1.02 (0.96-1.08)	0.49
**Respiratory rate at 2 hours after HFNC use (/min)$**	32 (30, 40)	41.5 (36.5, 55)	1.10 (1.03-1.18)	*0.006**
**Respiratory rate decreased at least 20% after HFNC use for 2 hours**	28 (93.3)	7 (35.0)	0.04 (0.01-0.21)	*<0.001**
**Heart rate at 0 hour (/min)$**	153 (138, 167)	166 (150, 180)	1.04 (1.01-1.07)	*0.028**
**Heart rate at 2 hours after HFNC use (/min)$**	119 (106, 130)	150 (140, 169)	1.09 (1.04-1.15)	*<0.001**
**Heart rate decreased at least 20% after HFNC use for 2 hours**	29 (96.7)	6 (30.0)	0.02 (0.002-0.14)	*<0.001**
**ROX# index at 0 hour$**	4.86 (4.30, 5.77)	4.65 (3.96, 5.61)	0.75 (0.43-1.32)	0.32
**ROX# index at 2 hours after HFNC use$**	6.72 (5.77, 8.22)	5.44 (4.21, 6.44)	0.48 (0.29-0.78)	*0.003**
**ROX# index increased at least 20% after HFNC use for 2 hours**	26 (86.7)	6 (30.0)	0.07 (0.12-0.27)	*<0.001**

#ROX index (**R**espiratory rate-**OX**ygenation) as the ratio of oxygen saturation/fraction of inspired oxygen to respiratory rate ($SpO_2/FiO_2/RR$)
$present as median (interquartile range, IQR)
**statistical significance p-value < 0.05*
